# The Role of Alpha-7 Nicotinic Acetylcholine Receptors in Pain: Potential Therapeutic Implications

**DOI:** 10.2174/1570159X22666240528161117

**Published:** 2024-05-29

**Authors:** Yu Tao, Yufang Sun, Xinghong Jiang, Jin Tao, Yuan Zhang

**Affiliations:** 1 Clinical Research Center of Neurological Disease, Department of Geriatrics, The Second Affiliated Hospital of Soochow University, Suzhou 215004, P.R. China;; 2 Department of Physiology and Neurobiology, Centre for Ion Channelopathy, Medical College of Soochow University, Suzhou 215123, P.R. China;; 3 Jiangsu Key Laboratory of Neuropsychiatric Diseases, Soochow University, Suzhou 215123, P.R. China;; 4 MOE Key Laboratory of Geriatric Diseases and Immunology, Suzhou Medical College of Soochow University, Suzhou 215123, P.R. China

**Keywords:** α7 nicotinic acetylcholine receptors, chronic pain, ligand-gated ion channels, peripheral nervous systems, pharmacology, therapeutic approaches

## Abstract

Chronic pain represents a prevalent and costly medical challenge globally. Nicotinic acetylcholine receptors (nAChRs), one type of ligand-gated ion channels found extensively in both the central and peripheral nervous systems, have emerged as promising therapeutic targets for chronic pain. Although there are currently no FDA-approved analgesics specifically targeting nAChRs, accumulating preclinical and clinical evidence suggest that selective ligands for alpha 7 (α7) nAChRs show potential for treating chronic pain, boasting a reduced incidence of side effects compared with other nicotinic receptor types. The recent structural resolution of human α7 nAChRs has confirmed their negative association with heightened pain, providing a valuable foundation for the development of targeted medications. This review presents a comprehensive overview, encompassing insights into the roles of α7 nAChRs derived from structural and functional studies, recent advancements in pharmacology, and investigations into their involvement in the pathophysiology of chronic pain. Moreover, the review addresses the variability in analgesic effects based on the type of receptor agonist and highlights the current research limitations. As such, this review offers potential therapeutic approaches for the development of innovative strategies for chronic pain management.

## 1 INTRODUCTION

Pain is an unpleasant sensory and emotional experience associated with or resembling actual or potential tissue damage [1]. It is among the most prevalent symptoms reported by patients in clinical settings and is often accompanied by distressing subjective sensations. Chronic pain is defined as pain that persists or recurs for more than 3 months after the resolution of an acute illness or injury, according to the International Association for the Study of Pain classification of chronic pain [2]. Epidemiological evidence indicates that approximately one-fifth of the global population experiences chronic pain, with particular prevalence among the elderly [3]. As a result of the adverse impact of pain, there is significant interest in identifying new drug targets and developing effective analgesics for current pain treatment and clinical research. Despite the variety of analgesics available, such as nonsteroidal anti-inflammatory drugs (NSAIDs) and opioids, pain management remains challenging due to the undesirable side effects associated with these medications. Numerous studies have demonstrated the involvement of acetylcholine receptors (AChRs) in the regulation of various pain types, including inflammatory pain, neuropathic pain, and cancer-related pain. Consequently, the development of agents targeting AChRs has emerged as a promising strategy for pain management.

**Fig. (1):**
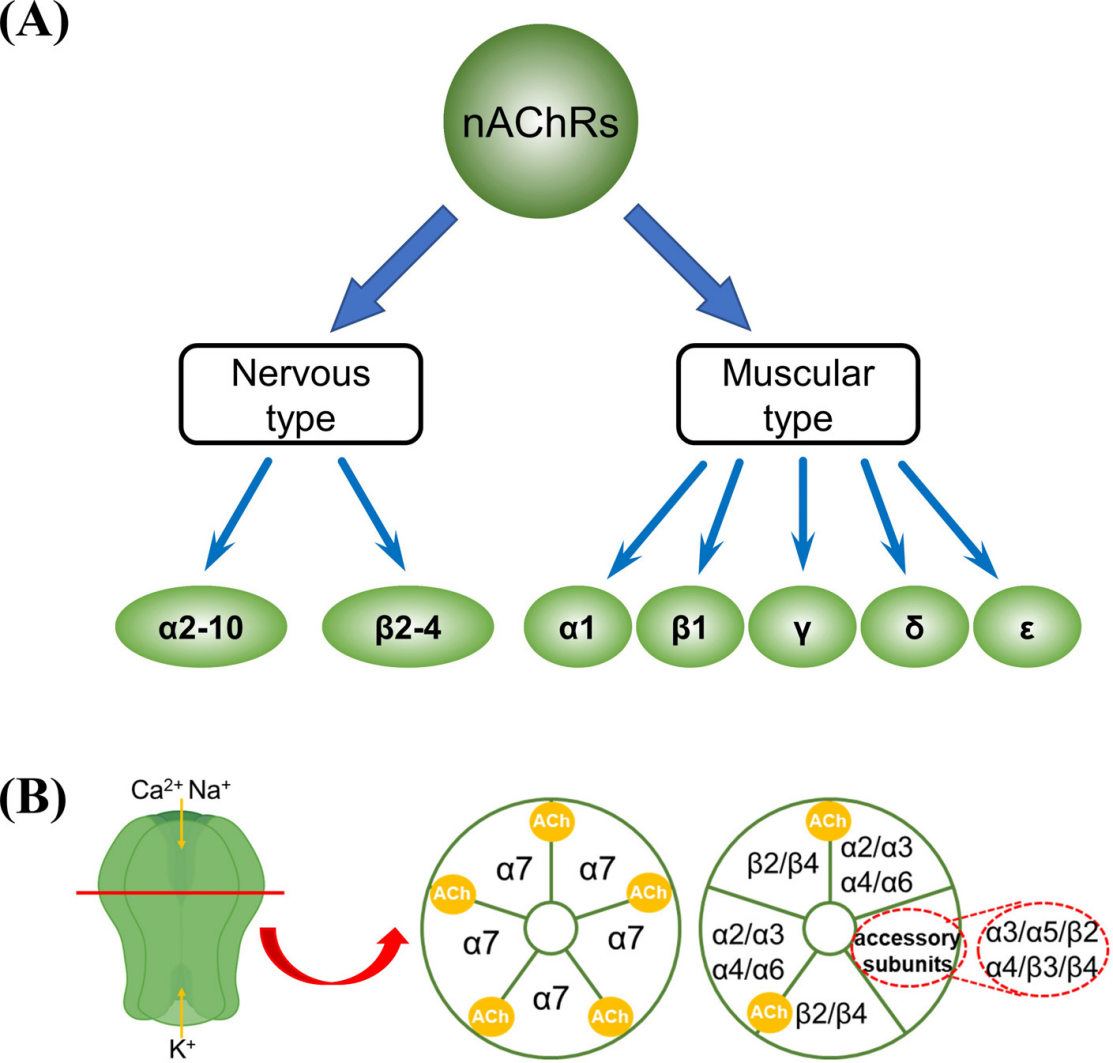
(**A**) Detailed classification of nicotine acetylcholine receptor subunits (nAChRs), which can be further categorized based on their distribution in either the muscular or nervous system. (**B**) Permutations of nAChR subunits in an α7 homomeric pentamer (*left*) and a heteromeric pentamer (*right*) in the central nervous system. The acetylcholines (AChs) shown in *yellow* represent the ligand binding sites on the nAChRs.

Generally, there are two main families of endogenous acetylcholine receptors located on the cell membrane: the nicotinic acetylcholine receptor (nAChR), which is a ligand-gated ion channel [4], and the muscarinic acetylcholine receptor (mAChR), which is a G protein-coupled receptor [5] (Fig. **1**). Nicotinic acetylcholine receptors are part of the Cys-loop superfamily of receptors and can be further categorized into two types: neuronal nAChRs and muscle-type nAChRs. Activation of neuronal nAChRs is associated with functions, such as memory, cognition, and sensory activity [6, 7], while muscle-type nAChRs are found at neuromuscular junctions and are the targets of muscle relaxants *in vivo* [8]. In mammals, a total of 17 subunits are known to be involved in the formation of various nAChR subtypes, including 10 α subunits (α1-α10), 4 β subunits (β1-β4), and 1 each of γ, δ, and ε subunits. The subunits of nAChRs exhibit distinct pharmacological characteristics, and the formed nAChR subtypes play different roles in polymerization and *in vivo* pathophysiological processes. These receptors can be categorized into two groups based on their affinity for ligands. One group includes receptors with a high affinity for vagus toxins, primarily the subtype containing α7, while the other group comprises receptors with a high affinity for acetylcholine and nicotine [9]. The latter receptors are predominantly αβ-type receptors, with the α4β2 receptor subtypes being dominant in the brain and the α3β4 receptor subtypes being dominant in the ganglia [10] (Table **1**).

The ligand-gated cation channel α7 nAChRs are widely distributed in both the central and peripheral nervous systems [11, 12]. Research has shown that α7 nAChRs play a crucial role in various physiological and pathological processes, including pain modulation, nicotine addiction [13], immunological homeostasis [14], and neurodegeneration [15]. Recent evidence strongly suggests that in peripheral sensory neurons, the heightened transmission efficiency due to α7 nAChRs leads to an amplification of sensory signals, particularly resulting in increased pain perception [16]. Despite these compelling findings, the precise contributions of α7 nAChRs to nociception and the sensitization of pain responses remain inadequately understood. This review aims to outline the most recent related research, including our own findings, which link the modulation of α7 nAChRs to pain processing and to summarize the modulation of their function by various exogenous compounds.

## 2 STRUCTURE AND CHARACTERISTICS OF THE α7 nAChRs

α7 nAChRs, as homopentamers, are often considered models for investigating the broader family of pentameric ligand-gated ion channels. These receptors open rapidly upon acetylcholine (ACh) binding, leading to cation influx and cell excitation [17]. Surprisingly, when considering macro currents, there is no significant difference in the single-channel current between α7 nAChRs with one functional ACh binding site and those with five functional ACh binding sites [18]. While the affinity of α7 nAChRs for traditional nAChR agonists like acetylcholine and nicotine is lower compared to other subtypes, their activation and desensitization are extremely rapid [19]. However, when measuring the net charge through the ion channel, the ability of acetylcholine to activate α7 nAChRs is not significantly different from that of other heteropentameric nAChRs [20-22]. Interestingly, the ion channel properties of α7 nAChRs may vary in different tissues. In nerves, the onset of desensitization by α7 nAChRs aligns closely with the opening and closing cycle, and the time course of discharge shows no concentration dependence on ACh, indicating that desensitization determines the rate of channel closing [23]. In contrast to muscle tissue, the desensitization onset time of α7 nAChRs is longer than the closing and reopening time, making it easier to distinguish between multiple open- and closed-state transitions of the same receptor channel. The switching cycle gradually shortens with increasing ACh concentration [24].

The ion channel properties of the α7 nAChR play pivotal roles in regulating a variety of biological processes, making them a fundamental characteristic to study. Electrophysiological markers serve as essential standards to be taken into account and evaluated throughout the entire process of developing ion channel drugs, from the research phase to clinical application. Consequently, investigating the ion channel properties of α7 nAChRs will provide valuable insights into their underlying mechanisms and prove advantageous in uncovering new opportunities for the development of related medications.

## 3 SIGNAL TRANSDUCTION OF THE α7 nAChRs

Membrane receptors serve as the interfaces for receiving and converting extracellular and intracellular signals, carrying significant biological importance. Calcium (Ca^2+^), a common second messenger, plays a crucial role in regulating the intracellular calcium signaling system by influencing various downstream signaling molecules, including calcineurin, adenylate cyclase, and tyrosine-protein kinase [25]. Interestingly, the effect of α7 nAChRs on intracellular calcium levels may be independent of α7 nAChR-mediated calcium influx [26] and instead rely on other ion channels regulated by α7 nAChRs, such as Cav1.2 [27]. Moreover, the activation of α7 nAChRs in inhibitory presynaptic terminals of the spinal cord significantly enhances the release of the inhibitory neurotransmitters GABA and glycine, which activate postsynaptic receptors and lead to substantial increases in the frequency of spontaneous and miniature inhibitory postsynaptic currents [28-30]. Specifically, targeting α7 nAChRs has been shown to reduce the accumulation of β-amyloid in the hippocampus, upregulate the calmodulin/Ca^2+^/calmodulin-dependent kinase II (CaMKII)/cyclic adenosine monophosphate (cAMP) pathway, and maintain synaptic morphology stability [31, 32]. Deletion of α7 nAChRs in the primary visual cortex has been observed to impair synaptic plasticity and potentially contribute to the onset of related disorders [33, 34].

Furthermore, advancements in sequencing technology have revealed that the *Chrna7* gene, which is responsible for encoding the α7 nAChR protein, exhibits high conservation across diverse species. However, variations exist in its modes and efficacies of action [35]. Given the multifaceted functions of α7 nAChRs, they stand as potential targets for therapeutic interventions across various diseases. The field of neuroscience has seen a growing emphasis in this area, leading to numerous breakthroughs in the development of specific targeted drugs.

## 4 TARGETING OF α7 nAChRs IN CHRONIC PAIN

Numerous studies have highlighted the involvement of α7 nAChRs in the regulation of chronic pain, encompassing inflammatory pain [36], neuropathic pain [37], and cancer pain [38]. Given the adverse effects and addictive nature of opioid receptor agonists, which have posed significant challenges for both patients and society [39], the exploration of new analgesic strategies, including the targeting of α7 nAChRs, holds promise for identifying alternative solutions to alleviate this issue. This avenue of research has the potential to uncover novel therapeutic targets for the clinical management of pain.

## 5 THE ROLE OF α7 nAChRs IN INFLAMMATORY PAIN

**Fig. (2):**
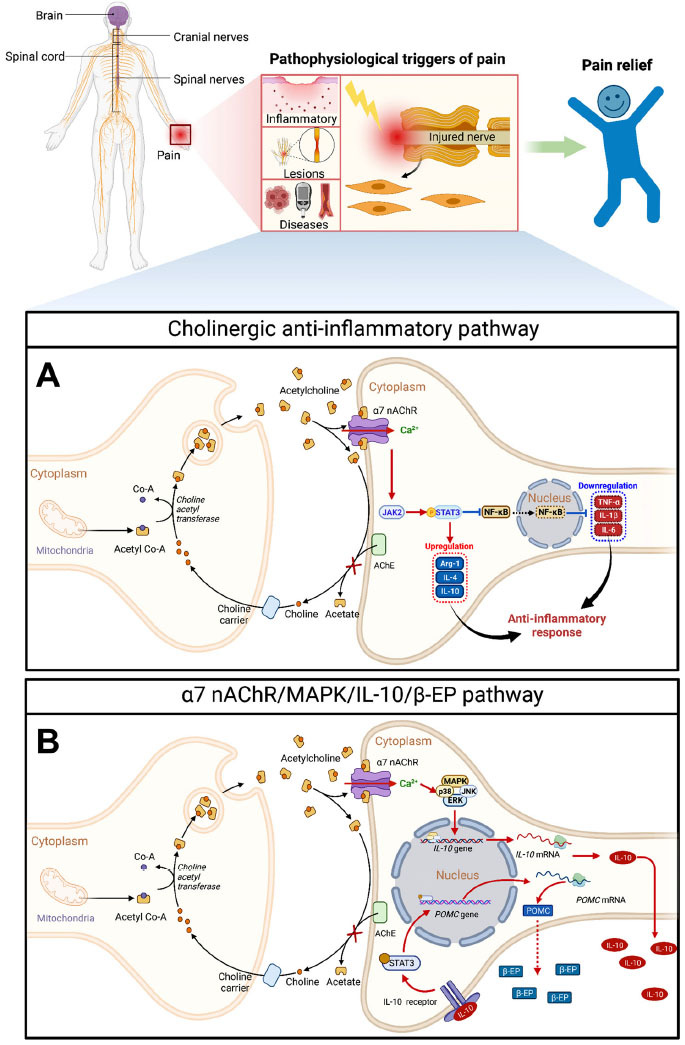
The schematic diagram presents the signaling pathways mediated by α7 nAChRs in chronic pain. (**A**) The cholinergic anti-inflammatory pathway is illustrated, wherein acetylcholine is synthesized in the presynaptic neuron from choline and acetyl coenzyme A (Co-A) and is then released into the synaptic space to facilitate neurotransmission. Acetylcholine binding to postsynaptic α7 nAChRs leads to their activation, initiating the JAK2 (Janus kinase 2)/STAT3 (signal transducer and activator of transcription 3) pathway. This activated pathway subsequently inhibits the nuclear translocation of nuclear factor kappa-B (NF-κB), down-regulates pro-inflammatory factors, including tumor necrosis factor (TNF-α), interleukin-1β (IL-1β), and interleukin-6 (IL-6), and up-regulates anti-inflammatory factors including arginase 1 (Arg-1), IL-4, and IL-10. (**B**) The α7 nAChR/MAPK (mitogen-activated protein kinase)/IL-10/β-EP (beta-endorphin) pathway is illustrated, where MAPK activation mediated by ligand-activated α7 nAChRs transcriptionally promotes the expression of IL-10. Secreted IL-10 then triggers the phosphorylation of STAT3 through IL-10 receptors. STAT3-mediated transcriptional activation up-regulates proopiomelanocortin (POMC), the precursor of β-EP, resulting in increased β-EP synthesis. The released β-EP, an endogenous opioid peptide highly effective in analgesia, activates μ-opioid receptors in neurons, thus inducing antinociception. Created with BioRender.com.

In typical scenarios, acute inflammation plays a crucial role in shielding the body against pathogens, as well as facilitating tissue remodeling and repair. However, when inflammation becomes chronic, persisting for 6 weeks or more, it can result in tissue damage and the sensation of pain. Inflammatory pain arises from the actions of numerous inflammatory mediators on nociceptive nerve endings, which heighten the sensitivity of additional receptors and ion channels, lower the excitability threshold of neurons, increase nerve discharge rates, and ultimately lead to the development of allodynia and hyperalgesia [40]. The cholinergic anti-inflammatory pathway, recognized for its substantial analgesic impact on inflammatory pain, was originally proposed by Borovikova [41]. Specifically, the vagus nerve serves as the conduit for conveying inflammatory stimulus signals to the central nervous system for detection and integration. Subsequent efferent signals *via* the vagus nerve exert their anti-inflammatory effects through the release of acetylcholine and the activation of specific receptors, particularly α7 nAChRs, on macrophages [42]. Upon activation by ligands, α7 nAChRs stimulate the JAK2/STAT3 pathway, which leads to the inhibition of nuclear factor kappa-B (NF-κB) nuclear translocation and the suppression of inflammasome activation [43]. This signaling cascade is implicated in downregulating proinflammatory factors, such as tumor necrosis factor (TNF-α), interleukin-1β (IL-1β), and interleukin-6 (IL-6) [44], while concomitantly upregulating anti-inflammatory factors, including arginase 1, IL-4, and IL-10 [45] (Fig. **2A**). Systemically administered α7 nAChR agonists appear to be preferentially effective in treating inflammation, which provides inspiration and a theoretical basis for researchers to focus on the effects of α7 nAChRs in the setting of inflammation. Studies have indicated that choline and α7 nAChR partial agonist GTS-21 exhibits a significant dose-dependent antinociceptive effect on postoperative inflammatory pain in an incisional model, potentially attributable to the inhibition of TNF-α by α7 nAChRs [46, 47]. The release of proinflammatory cytokines sensitizes the neurokinin-1 receptor (NK1R), which responds to substance P and other nociceptive substances, yet fails to have a significant effect on model mice following the knockout of the α7 nAChR [46]. Arthritis, a degenerative joint disease causing chronic disability in elderly individuals, often leads to severe pain during joint movement [48]. α7 nAChRs have also been found to be involved in the sensation of this pain [49, 50]. In the osteoarthritis (OA) model induced by sodium iodoacetate, activated α7 nAChRs have been shown to inhibit the expression of the OA marker matrix metalloproteinase-9 (MMP-9) and the translocation of NF-kB from the cytoplasm to the nucleus by enhancing the phosphatidylinositol 3-kinase (PI3K)/serine/threonine-protein kinase (Akt) signaling pathway, thereby alleviating pain [51].

Additionally, choline has been demonstrated to reduce pain resulting from rheumatoid arthritis by activating nitric oxide/cGMP/ATP-sensitive potassium channels and increasing K^+^ influx, resulting in neuronal hyperpolarization without interfering with inflammatory events, sedation, or motor impairment [52]. Cobratoxin has also been shown to reverse the increase in the serum levels of TNF-α, IL-1, and IL-2 and the decrease in the serum levels of IL-10 in complete Freund’s adjuvant (CFA)-induced OA model rats, with these effects being blockable by methyllycaconitine (MLA) [53].

Interestingly, contrary to prominent reports, the long-chain neurotoxic protein alpha-cobratoxin (α-CTx) is considered an antagonist binding with high affinity to α7 nAchRs in the peripheral and central nervous systems [54]. However, behavioral tests have reported α-CTx-induced antinociceptive effects to be antagonized by atropine, a nonselective mAChR antagonist [55]. These findings have been further confirmed by recent studies using an extracellular electrophysiological approach [56, 57], indicating the involvement of M3 and M4 mAChRs, not merely α7 nAchRs, in the analgesic effects of these agents.

The analgesic effects of α7 nAChRs have been reported in various models of inflammatory pain beyond OA, but the underlying mechanisms of action may vary. In a dextran sodium sulfate-induced colitis model, α7 nAChR agonists were shown to effectively reverse mechanical hyperalgesia [58]. However, it is important to note that while analgesic effects were observed, there were no significant improvements in colon injury or inflammation. This finding suggested that the role of α7 nAChRs in pain modulation may be distinct from their effects on inflammation in this particular model [59, 60]. Bagdas *et al*. conducted a study comparing the effects of the GAT107, which is not only an α7 nAChR positive allosteric modulator (PAM) but also an α7 nAChR allosteric agonist [61], in various inflammatory pain models. They administered these compounds through plantar and intrathecal injections. They reported that the antihyperalgesic effect of these compounds occurs primarily through the central nervous system rather than the peripheral nervous system [62]. Importantly, their research provided new insights into the role of α7 nAChRs in pain regulation. They investigated the stretching behavior and conditioned place aversion behavior of mice injected with acetic acid in the abdominal cavity and discovered that the antinociceptive effect of α7 nAChRs is associated with the regulation of negative emotion rather than sensation [63]. However, the anti-inflammatory effects of α7 nAChRs remain contentious, potentially due to their specificity for certain organs and diseases. A rigorous study demonstrated that the activation of α7 nAChRs does not have anti-inflammatory or antinociceptive effects on all inflammatory joint diseases. These researchers confirmed this through experiments on the CFA-induced monoarthritis model, showing that α7 nAChR activation exacerbates joint inflammation and pain, potentially involving mast cells that regulate neuroimmune mechanisms [64].

Studies on the role of α7 nAChRs in inflammatory pain have extended beyond neurons to include nonneuronal cells. Specifically, the expression of α7 nAChRs in microglia and astrocytes has been observed and confirmed through immunofluorescence staining [65]. It has been proposed that following peripheral nerve injury, glial cells, particularly microglia, undergo various morphological and functional changes. Excessive activation of microglia leads to the production of inflammatory factors and nerve damage. However, activation of α7 nAChRs on microglia can mitigate nerve damage caused by inflammation and oxidative stress through multiple anti-inflammatory pathways [66]. Activation of α7 nAChRs on microglia in the hippocampus has been shown to reduce the expression of the NF-κB inhibitory protein and CD11b in an LPS-induced inflammatory pain model. This process promotes microglial activation and helps alleviate hyperalgesia [67, 68].

Additionally, it has been argued that continuous stress-induced release of proinflammatory cytokines in the hippocampus can be reversed by increasing the expression of α7 nAChRs through vagus nerve stimulation. This improvement in α7 nAChR expression helps ameliorate the pain response and depressive-like behavior [69]. In formalin-induced inflammatory pain, α7 nAChRs can exert antihyperalgesic effects through the IL-10/β-endorphin pathway in spinal microglia [70] (Fig. **2B**). Astrocytes, despite being mostly associated with neurodegenerative diseases, also express α7 nAChRs to some extent [71]. In neuroinflammatory mouse astrocytes cultured *in vitro*, activation of α7 nAChRs can reduce inflammation and oxidative stress, exerting neuroprotective effects that may also be relevant to pain management [72]. In nonneuronal cells, α7 nAChRs can also play an anti-inflammatory and analgesic role. For instance, the selective silent agonist of α7 nAChRs reduces the number of proinflammatory bone marrow-derived monocytes/macrophages and alleviates mechanical hypersensitivity induced by CFA by inhibiting monocyte viability and proliferation [73]. As a result, targeting α7 nAChRs on glial cells could be a valuable approach for attenuating inflammatory pain.

## 6 THE ROLE OF α7 nAChRs IN NEUROPATHIC PAIN

Neuropathic pain is characterized by heightened sensitivity to pain and the presence of spontaneous pain due to lesions or diseases involving the somatosensory nervous system [74]. In the pathological state of neuropathic pain, various ion channels, membrane receptors, and signaling molecules on neurons undergo changes to varying degrees [75]. In the chronic constriction injury (CCI) model, which is a commonly used neuropathic pain model, the pain threshold of mice lacking α7 nAChRs shows little change, whereas mice with increased expression of α7 nAChRs demonstrate significant improvement in thermal and mechanical hypersensitivity [76]. These findings suggest that α7 nAChRs may serve as a potential target for the treatment of neuropathic pain. Further research on the CCI model revealed that activation of α7 nAChRs led to a significant decrease in the activity of activation transcription factor 3 (ATF3), a marker of neuronal damage. However, the number of positive cells did not show significant changes [77].

Furthermore, the activation of phosphorylated extracellular signal-regulated kinase (pERK) and satellite cells in the dorsal root ganglia of rats with CCI was significantly inhibited, leading to the effective alleviation of mechanical hypersensitivity [77]. Purine nucleotides, which are important extracellular regulators of pain, participate in pain modulation by activating purinoceptors, including the P2X (ionotropic) and P2Y (metabotropic) receptors [78]. Among the P2Y12 receptor antagonists, PSB-0739 showed the strongest effect on pain when administered intrathecally and significantly increased the mechanical pain threshold in the partial sciatic nerve ligation model. However, methyllycaconitine (MLA), a specific α7 nAChR antagonist, was able to block the effects of PSB-0739 on pain behavior and cytokine expression, indicating that the P2Y12 receptor pathway involves α7 nAChR-mediated efferent pathways [79]. The cDNA library constructed based on genes derived from rat dorsal root ganglia on day 14 after peripheral axotomy revealed significant changes in the expression of the α7 nAChR and P2Y1 receptors [37]. These findings further support the idea that P2Y receptors and α7 nAChRs are involved in the coregulation of neuropathic pain and highlight their importance as therapeutic targets for managing this condition.

The recent discovery of the novel acetylcholine receptor chaperone (NACHO) has shed light on a unique molecular chaperone present exclusively in neurons. Its crucial role lies in facilitating the assembly and transportation of α7 subunits. To investigate the impact of NACHO deficiency on congenital functional expression, mice with a *tmem35a* gene knockout (responsible for NACHO production) were utilized. This model successfully simulated the loss of *Nacho* gene function. The findings revealed an enhanced neuroinflammatory response in the spinal cord, evident through heightened mechanical and thermal sensitivity. However, despite the absence of NACHO, the administration of α7 nAChR-specific agonists through intrathecal injection still managed to alleviate hypersensitivity [80].

Furthermore, in a migraine rat model, it was observed that the expression of the α7 nAChR in the hippocampus was diminished. This reduction in the α7 nAChR led to the activation of microglia and astrocytes, resulting in the release of TNF-α, IL-1β, and calcitonin gene-related peptide (CGRP) through the downstream p-JNK/MAPK signaling pathway. Consequently, the rats experienced hyperalgesia and hypersensitivity. However, these effects were significantly alleviated by intracerebral injection of the α7 nAChR agonist PNU-282987 [81]. Melatonin, a hormone secreted by the pineal gland, not only regulates the circadian rhythm and possesses antioxidant properties but has also been found to have remarkable analgesic effects with minimal side effects on chronic pain, including migraines [82, 83]. In cultured rat glioma C6 cells, melatonin has shown the ability to dose-dependently downregulate LPS-induced inflammatory factor release and high expression of α7 nAChRs [84]. Additionally, in migraine models, the neuroprotective effect of melatonin can be blocked by specific antagonists of α7 nAChRs, suggesting a pivotal role of α7 nAChRs in the regulation of migraines [85].

Microglia can be divided into two polarization states: the proinflammatory M1 type and the anti-inflammatory M2 type. The M1 type primarily releases TNF-α, IL-1β, and IL-6, while the M2 type mainly secretes brain-derived neurotrophic factor, glial cell-derived nutritional factor, and IL-10 [86]. Normally, these two types maintain a dynamic balance. However, after nerve injury, microglia are more likely to transition from a resting state to the M1 type, producing proinflammatory factors that play a crucial role in the initial stage of neuropathic pain. For instance, in a rat model of neuropathic pain, the majority of the activated microglia in the posterior horn of the spinal cord on the 7^th^ and 14^th^ days following CCI were of the M1 type, potentially contributing to pain development [87]. In a sciatic nerve ligation model, increased expression of dynorphin A leads to the release of IL-1β and TNF-α, promoting hyperalgesia. However, these effects can be reversed by intrathecal injection of α7 nAChR agonists or microglial inhibitors [88]. *In vitro* cultures of primary rat microglia have shown that cynandione A upregulates IL-10 and β-endorphin, but does not affect α7 nAChRs [89, 90]. In the L5/L6 spinal nerve ligation model, cynandione A activates the AMP/PKA/p38/CREB signaling pathway through α7 nAChR-dependent phosphorylation of spinal microglia.

Additionally, it promotes the expression of β-endorphin *via* the IL-10/STAT3 signaling pathway, resulting in an antihyperalgesic effect [89]. Lemairamin, an agonist of α7 nAChRs found in Zanthoxylum plants, also demonstrates an antinociceptive effect through this pathway. However, this effect can be blocked by intrathecal injection of the microglial activation inhibitor minocycline, an IL-10 neutralizing antibody, or anti-β-endorphin serum [91, 92].

## 7 THE ROLE OF α7 nAChRs IN CANCER PAIN

The quality of life for cancer patients is significantly impacted by severe pain caused directly or indirectly by primary or metastatic cancer. Therefore, it is crucial to investigate methods to alleviate pain in these patients [93]. Studies have indicated that the α7 nAChR present in tumor-associated macrophages has the ability to inhibit tumor metastasis *via* the JAK2/STAT3 signaling pathway [94]. Additionally, the overexpression of α7 nAChR in human colorectal cancer LoVo cells can suppress tumor invasion through the PI3K/ Akt signaling pathway [95].

In rats with cancer-induced bone pain, the majority of α7 nAChRs in the spinal cord are located in neurons. However, their expression significantly decreases on the 21^st^ day after surgery, leading to a reduction in the paw withdrawal threshold (PWT) to a minimum of less than 4 g [38]. Cinobufagin, a bioactive compound found in bufanolide steroids, is known for its pain-relieving and anti-inflammatory properties and is commonly used in antitumor treatments [96]. Studies have shown that rats with bone cancer exhibit noticeable mechanical allodynia due to a significant reduction in the expression levels of IL-10 and β-endorphin in spinal cord microglia [97]. The activation and expression of IL-10 and β-endorphin in spinal microglia, mediated by cinobufagin or the α7 nAChR-selective agonist PHA-543613, can be blocked by the α7 nAChR-specific antagonist MLA, which inhibits the analgesic effects [97].

Furthermore, studies have revealed that the expression of α7 nAChR in the spinal cord decreases in rats with bone cancer, leading to activation of the NF-κB pathway, which contributes to the development of bone cancer pain. However, intrathecal injection of PNU-282987, an α7 nAChR agonist, can inhibit the NF-κB pathway and provide relief from hyperalgesia [38]. Intrathecal injection of lemairamin, another α7 nAChR agonist, dose-dependently alleviates mechanical allodynia in the ipsilateral hind paw (but not the contralateral hind paw). This effect is achieved by activating spinal α7 nAChRs and mediating the IL-10/β-endorphin pathway. These findings have been confirmed in rats with Walker 256 carcinoma cells implanted in the tibial cavity [38].

Cancer pain can arise not only from the cancer itself but also from various medical treatments and procedures. One such treatment is oxaliplatin, a third-generation platinum antitumor drug that can induce neurotoxicity in the dorsal root ganglia, leading to hyperalgesia [98]. Studies have demonstrated a significant reduction in the expression of α7 nAChR in both the peripheral and central nervous systems of rats treated with intraperitoneal injections of oxaliplatin [99]. To counteract oxaliplatin-induced pain and protect nerve tissue, α7 nAChR agonists like (R)-ICH3 and PNU-282987 have been found to be effective. These agonists can increase the density of astrocytes in the spinal cord and somatosensory area while inhibiting pain caused by oxaliplatin [99, 100]. Activation of the α7 nAChR promotes the expression and release of the anti-inflammatory factor TGF-β1 and the glutamate-detoxifying enzyme glutamine synthetase. It also activates astrocytes, maintaining a physiological balance between neurons and glia, thus relieving the neurotoxicity induced by oxaliplatin and mediating pain relief [101]. Preclinical studies have demonstrated that CDP-choline when administered intracerebroventricularly, can target α7 nAChRs and exert an antihyperalgesic effect induced by oxaliplatin. This effect is achieved through the involvement of GABA receptors and opioid receptors [102, 103]. The mammalian target of the rapamycin complex plays a crucial role in cell proliferation, growth, and survival, and its natural inhibitor, rapamycin, is commonly used as an anticancer agent [104]. However, rapamycin has been found to induce hyperalgesia in animal models [105]. When microinjected into the anterior cingulate cortex (ACC), rapamycin has been shown to phosphorylate various substrates, such as insulin receptor substrate-1, Akt, and ERK in this region. Nicotine has the ability to counteract the effects of rapamycin, inhibit the firing frequency of ACC neurons, and alleviate pain. However, the antihyperalgesic effect of nicotine can only be partially blocked by α4β2 or α7 nAChR blockers [105] because of the simultaneous activation of various nAChRs in different tissues with systemic administration of nicotine.

Additionally, systemic administration of nicotine activates various nAChRs in different tissues, so blocking α7 nAChRs can only partially hinder the analgesic effect of nicotine. In conclusion, α7 nAChRs play a role in the hyperalgesia caused by antitumor drugs. Excitingly, efforts are being made to alleviate the pain experienced by cancer patients during treatment by targeting this receptor.

## 8 THE ROLE OF α7 nAChRs IN OPIOID-INDUCED HYPERALGESIA

To date, the use of opioids such as fentanyl, morphine, remifentanil, and sufentanil remains prevalent in the treatment of acute and chronic pain, as well as during anesthesia for surgery. However, long-term or high-dose opioid use can lead to drug addiction and the development of drug resistance [106]. When opioids are discontinued, patients may experience hyperalgesia, known as opioid-induced hyperalgesia (OIH). Research has shown that prolonged opioid exposure can lead to a loss of μ receptors and the activation of the glutamatergic system, resulting in heightened sensitivity to pain [107]. OIH not only increases the requirement for postoperative analgesics but also exacerbates postoperative discomfort and significantly impacts patient recovery. Therefore, the clinical use and dosage of opioids need to be carefully regulated. Studies have demonstrated that the combined administration of the α4β2 nAChR agonist A85380 with fentanyl can not only reduce the side effects of fentanyl, such as dyspnea and apnea, but also enhance its antihyperalgesic effects [108]. This approach has proven to be an effective pain treatment strategy. Based on this, it can be hypothesized that α7 nAChRs may play a similar role in OIH. The α7 nAChR agonists PHA-543613 and the PAM PNU-120596 have shown significant increases in mechanical and thermal withdrawal thresholds in rat models of remifentanil-induced pain [109]. Indeed, studies have indicated that α7 nAChRs are involved in OIH, although the specific underlying mechanism is still not fully understood. Research has shown that α7 nAChR allosteric activators can effectively alleviate postoperative hyperalgesia induced by remifentanil in rats [109]. Activation of α7 nAChRs in the spinal cord can inhibit excessive activation of microglia and astrocytes, thereby reducing the expression of brain-derived neurotrophic factor (BDNF), TNF-α, IL-6, and TrkB while increasing the level of KCC2, ultimately relieving hyperalgesia [110]. This mechanism is thought to involve the inhibition of phosphorylated NR2B (p-NR2B) and BDNF expression at the spinal cord level, which subsequently helps to suppress the excitatory signaling pathway. Activation of α7 nAChRs results in the enhancement of the inhibitory effect, thus participating in the regulation of OIH [109, 111]. Additionally, in the context of burn injury, repetitive opioid use can exacerbate pain at the burn injury site, while the α7 nAChR agonist GTS-21 shows potential in treating both morphine-induced aggravated burn injury pain and microglial activation [112].

Combination therapy is a widely employed clinical treatment strategy aimed at enhancing the efficacy of drugs, reducing the occurrence of drug resistance, and minimizing the toxicity and side effects of drugs. However, it is important to acknowledge that this approach may occasionally yield undesired outcomes. Therefore, a thoughtful and well-informed selection of drug combinations necessitates further research and a thorough understanding of the interactions between different drugs, with careful consideration given to the avoidance of adverse reactions. To explore the potential benefits, the combination of opioids with α7 nAChR agonists has been investigated for this purpose.

## 9 THE TREATMENT OF PAIN BY SPECIFIC AGONISTS AND PAMs OF α7 nAChRs

As mentioned previously, the adverse effects associated with common opioid analgesics, particularly addiction, have prompted scientists to explore alternative options for effective pain relief with fewer drawbacks. Among the nAChRs, the α7 subtype has garnered significant attention due to its widespread distribution, abundant expression, and notable involvement in pain modulation. Specific agonists targeting α7 nAChRs have demonstrated remarkable analgesic effects and have undergone clinical trials. However, prolonged use of exogenous α7 nAChR agonists can impact receptor expression, potentially leading to excessive receptor activation, desensitization, and diminished drug efficacy [113]. Positive allosteric modulators (PAMs) offer a solution to this issue, as they can bind to α7 nAChRs without agonistic activity. Instead, they induce conformational changes that facilitate the binding of endogenous ligands, thereby enhancing their effects. PAMs achieve this by reducing the energy barrier required for the receptor to transition from a resting state to an activated state [114]. Generally, PAMs can be classified into two categories: type I PAMs (*e.g*., LY-2087101 and NS1738) enhance agonist effects without impacting desensitization, while type II PAMs (*e.g*., PNU-282987 and PNU-120596) not only possess antihyperalgesic properties but also inhibit desensitization and prolong receptor activation [115]. The efficacy of these PAMs, along with certain noncanonical agonists, may vary in animal models [116]. For instance, in the carrageenan model, both types of drugs can alleviate thermal hyperalgesia. However, PNU-120596 has the additional benefit of eliminating local edema symptoms, which NS1738 cannot achieve. In the CCI model, PNU-120596 exhibits a long-lasting antihypersensitivity effect compared to NS1738 [117]. Therefore, researchers are focusing on the discovery and synthesis of PAMs targeting α7 nAChRs that offer increased specificity, improved analgesic effects, reduced side effects, and lower manufacturing costs.

Interestingly, certain ingredients from traditional Chinese medicine, such as curcumin found in turmeric, act as PAMs for α7 nAChR and have demonstrated the ability to alleviate tonic and visceral pain in mice [118, 119]. Furthermore, a newly developed drug, known as the specific silent agonist of α7 nAChR, has been tested for its pain-relieving properties [120]. When used alone, a silent agonist can rapidly induce a stable state of desensitization in combination with α7 nAChRs, which differs from the traditional agonists [121]. In this state, only a small portion of α7 nAChRs are activated, generating no significant current. As a result, α7 nAChRs exhibit higher sensitivity to type II PAMs compared to type I PAMs, making certain non-specific type II PAMs more selective for α7 nAChRs and enhancing their efficacy [122, 123]. For example, the selective α7 nAChR silent agonist NS6740 has shown excellent results in reversing pain thresholds and effectively controlling the emotional aspects of pain in nociceptive behavioral models induced by formalin, acetic acid, and CCI [120].

Furthermore, drugs targeting α7 nAChRs have shown potential in inhibiting hyperalgesia when coadministered with other drugs. In addition to the beneficial effects observed with the combined administration of α7 nAChR agonists and opioid receptor agonists mentioned earlier, the combination of α7 nAChR agonists and aspirin has also demonstrated unexpected effects [124]. The combined administration of choline at a nonalcoholic dose of 8 mg/kg and aspirin at a low analgesic dose of 5 mg/kg resulted in a faster onset time and longer duration of action compared to aspirin alone [124]. The use of MLA, an α7 nAChR antagonist, was found to block the antinociceptive effect of choline in a mouse model of visceral inflammatory pain induced by acetic acid and carrageenan. These findings indicate that the α7 nAChR is a critical target for this combination treatment and provides a basis for developing novel analgesic therapies with lower doses, longer treatment durations, and fewer side effects [125].

Interestingly, in a human study, oral supplementation with choline perioperatively did not appear to reduce pain or opioid requirements, possibly due to the lack of a significant increase in plasma choline levels [126]. However, two other preclinical studies demonstrated that inflammatory pain could be alleviated through intracerebroventricular or intraplantar administration of CDP-choline [127, 128]. These findings suggest that selecting an appropriate administration route for precise drug delivery may effectively enhance the efficacy of cholinergic drugs.

**Fig. (3):**
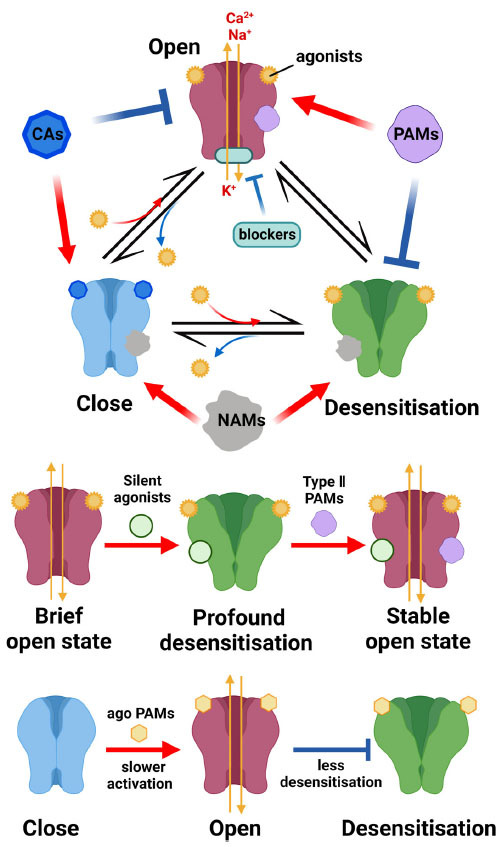
Different types of drugs can interact with α7 nicotinic acetylcholine receptors (α7 nAChRs) through various mechanisms, resulting in different effects on receptor activity. Positive allosteric modulators (PAMs) or negative allosteric modulators (NAMs) can bind to regulatory sites to cause conformational changes in α7 nAChRs to activate or inhibit the effects, respectively. Competitive antagonists can bind to the active sites of α7 nAChRs without causing biological effects or blocking the effects of agonists. Open-channel blockers can directly block ions from passing through α7 nAChRs. Silent agonists produce little functional agonist activity but induce a type II PAM-sensitive desensitized state. In this profound desensitized state, α7 nAChR could be strongly activated by type II PAMs, which in turn provides further stabilization to the open channel. Allosteric agonists (ago-PAMs) are able to activate α7 nAChRs in the absence of an orthosteric agonist and typically display a slower activation and induce considerably less desensitisation than orthosteric agonists. Created with BioRender.com.

Currently, the research studies on analgesic drugs targeting α7 nAChRs have primarily focused on the agonistic effects of α7 nAChRs, with limited studies on the application of antagonists. However, apart from their potential in pain relief, preclinical experiments have demonstrated the promising efficacy of α7 nAChR agonists and positive allosteric modulators (PAMs) in treating complex and challenging diseases, such as Alzheimer’s disease, epilepsy, Parkinson’s disease, and schizophrenia [32, 129]. Notably, competitive antagonists, negative allosteric modulators (NAMs), and open-channel blockers targeting α7 nAChRs have also shown usefulness (Fig. **3**). Given the extensive application of α7 nAChR-related drugs and the wide target population, the development of such drugs holds significant importance and requires increased attention.

## CONCLUSION

The remarkable antihyperalgesic and antiallodynic effects of α7 nAChRs on different types of pain suggest a promising avenue for the development of analgesic drugs [130] (Table **2**). Existing experimental data have primarily focused on investigating the characteristics of ion channels, downstream signaling pathways under pathological conditions, and the synthesis and effects of specific chemical drugs in relation to the role of α7 nAChRs in pain treatment. However, the modulation of the downregulation of α7 nAChRs observed in pathological pain by upstream molecules remains an area worth investigating. Overcoming the challenge of rapid desensitization of α7 nAChRs to agonists has proven to be difficult. For instance, oral administration of exogenous agonists like choline has shown limited efficacy and has fallen short of expectations [126]. Consequently, exploring alternative mechanisms that differ from agonists and positive allosteric modulators (PAMs) to reverse the changes in α7 nAChR expression upstream may be crucial for achieving long-term stable analgesic effects through safe and efficient administration methods.

Recent experiments have provided evidence that, alongside the α7 homopentamer found in natural neurons, there exists a smaller population of functional heteropentamer α7β2 nAChRs formed by α7 and β2 subunits [131]. Electrophysiological analyses have revealed that α7β2 nAChRs exhibit distinct characteristics when compared to traditional α7 nAChRs. These differences include slower decay kinetics of the whole-cell current and lower amplitudes of the whole-cell current [131]. Interestingly, the specific characteristics of α7β2 nAChRs vary depending on the number and position of β2 subunits inserted [132]. For instance, the insertion of one β2 subunit at position 3 results in a higher current amplitude compared to the insertion of two β2 subunits at positions 2 and 4 [132]. These findings raise two important questions: firstly, whether α7β2 nAChRs are involved in chronic pain pathways, and secondly, whether analgesic drugs targeting α7 nAChRs also have effects on α7β2 nAChRs. Further researches are needed to explore these questions and the potential implications for chronic pain treatment and the use of analgesics.

Apart from the role of α7 nAChRs in pain modulation, other subtypes of nAChRs also contribute to cholinergic-related signal regulation in diverse ways. While much previous research has focused on α4β2 nAChRs, the specific side effects of these drugs are substantial, and their application is limited. As a result, scientists are now exploring more advantageous analgesic drugs targeting the α6β4, α7, or α9-containing subtypes. Moreover, the patterns of changes in different nAChR subtypes in pain models vary. For instance, in various chronic pain pathologies, the expression of α6β4 nAChRs and α7 nAChRs is reduced [111, 133], while the expression of α9 subunits is increased [134]. This finding reminds us that research on nAChRs cannot be generalized, and there may be coordination or antagonism between different subtypes; however, there are still many gaps in related research.

Overall, the role of α7 nAChRs in pain modulation is crucial and should not be overlooked, and the underlying molecular regulatory mechanism requires further detailed study. In addition, Analgesic drugs targeting α7 nAChRs have significant effects, few side effects, diverse types, and high demand. However, most of these drugs are used in preclinical research, and further studies with larger sample sizes are needed to enhance their clinical application value.

## Figures and Tables

**Table 1:** Summary of main subtypes nAChRs in pain.

**Subtypes**	**Tissue-specific Expression**	**Brain Regions Location**
α4β2 nAChRs	Brain, liver, parathyroid gland, retina	Cerebral cortex, pons and medulla, basal ganglia, midbrain, olfactory region
α5-containing nAChRs	Brain, smooth muscle, gallbladder	Hippocampus, cerebral cortex, hypothalamus
α6β4 nAChRs	Brain, retina, adrenal gland, thymus	Cerebral cortex, cerebellum, midbrain, pons and medulla, amygdala
α7 nAChRs	Small intestine, brain, adrenal gland, stomach, retina (neuron, astrocyte, macrophage, microglia, cancer cell, *etc*.)	Pons and medulla, cerebral cortex, hypothalamus, hippocampus, amygdala
α9α10 nAChRs	Skeletal muscle, brain, pituitary gland, blood	Cerebral cortex, pons and medulla, amygdala, hypothalamus

**Table 2:** Summary of studies on α7 nAChR in pain.

**Types of Pain**	**Model**	**Sample**	**Agonist and PAM ** **(Administration Route)**	**References**
Inflammatory pain	Postoperative inflammatory pain	Human blood	Choline (p.o.)	[126]
		Mouse macrophage	Choline (s.c.)	[46]
		Mouse superficial/deep dorsal horn	Nicotine/GTS-21 (i.t.)	[47]
		Rat spinal cord/spinal microglia	PHA-543613/PNU-120596 (i.t.)	[70]
-	Osteoarthritis	Mouse tibiofemoral joint/RAW 264.7 cells	Nicotine (i.p.)	[51]
		-	Choline (i.p./i.t.)	[49]
		Rat serum	Cobratoxin (i.p.)	[53]
		Mouse periarticular tissue/HMC-1	AR-R17779/ A844606 (i.p.)	[64]
	Colitis	Mouse colon	Nicotine (p.o.)/PNU-282987 (i.p.)	[58]
	Paw inflammation pain	Mouse spinal cord	GAT107 (i.p./i.t.)	[62]
	Inflammatory substances (i.p.)	Mouse hippocampus	TQS (i.p.)	[67, 68]
	Myalgic encephalomyelitis	Mouse gastric and hippocampal tissue	-	[69]
Neuropathic pain	CCI	*CHRNA7* knockout/knockin mouse	-	[76]
		Rat spinal cord/DRG	TC-7020 (i.t.)	[77]
	SNL	Rat spinal cord	-	[79]
		Rat spinal cord/primary spinal microglia	PHA-543613 (i.t.)	[97]
		Rat spinal cord	Lemairamin (i.t.)	[92]
		Rat spinal cord/primary spinal microglia	Cynandione A (i.t.)	[89]
	*tmem3a* knockout	Mouse spinal cord	PHA543613 (i.t.)	[80]
	Chronic migraine	Rat hippocampus	PUN-282987 (i.c.v.)	[81]
Cancer pain	Cancer-induced bone pain	Rat spinal cord	PUN-282987 (i.t.)	[38]
		Rat spinal cord/primary spinal microglia	Cinobufagin (i.t.)	[97]
		Rat spinal cord	Lemairamin (i.t.)	[92]
	Cancer treatment-induced pain (oxaliplatin/rapamycin)	Rat sciatic nerves/DRG/spinal cord	(R)-ICH3/PNU-282987 (p.o.)	[99]
		Neurons and astrocytes	PNU-282987	[101]
		-	CDP-choline (i.c.v.)	[103]
		Mouse ACC	Nicotine (a.c.c)	[105]
Opioid-induced hyperalgesia	Fentanyl-induced hyperalgesia	Rat brainstem-spinal cord and medullary slice	Nicotine/PNU-282987 (s.c.)	[108]
	Remifentanil-induced hyperalgesia	Rat spinal cord	PHA-543613/PNU-120596 (i.t.)	[109]
		Rat spinal cord	PNU-120596 (i.t.)	[110]

**Abbreviations**: p.o., per os; s.c., subcutaneous injection; i.t., intrathecal injection; i.p., intraperitoneal injection; i.c.v., intracerebralventricular injection; a.c.c., anterior cingulate cortex injection; RAW 264.7 leukemia cells in mouse macrophage; HMC-1, human mast cell-1 line.

## LIST OF ABBREVIATIONS

AChRs: Acetylcholine Receptors
ATF3: Activation Transcription Factor 3
cAMP: Cyclic Adenosine Monophosphate
CCI: Chronic Constriction Injury
IL-6: Interleukin-6
mAChR: Muscarinic Acetylcholine Receptor
MLA: Methyllycaconitine
MMP-9: Marker Matrix Metalloproteinase-9
nAChR: Nicotinic Acetylcholine Receptor
NF-κB: Nuclear Factor Kappa-B
NSAIDs: Nonsteroidal Anti-inflammatory Drugs
OIH: Opioid-induced Hyperalgesia
PAM: Positive Allosteric Modulator
TNF-α: Tumor Necrosis Factor
